# Interactions between the Entomopathogenic Fungus *Metarhizium anisopliae* ICIPE 20 and the Endoparasitoid *Dolichogenidea gelechiidivoris*, and Implications for Combined Biocontrol of *Tuta absoluta*

**DOI:** 10.3390/biology11091323

**Published:** 2022-09-06

**Authors:** Sahadatou Mama Sambo, Komivi Senyo Akutse, Hannalene du Plessis, Pascal Osa Aigbedion-Atalor, Samira Abuelgasim Mohamed, Shepard Ndlela

**Affiliations:** 1 International Centre of Insect Physiology and Ecology (*icipe*), Nairobi P.O. Box 30772-00100, Kenya; 2 Unit for Environmental Sciences and Management, North-West University, Private Bag X6001, Potchefstroom 2520, South Africa; 3 Centre for Biological Control, Department of Zoology and Entomology, Rhodes University, Makhanda 6140, South Africa

**Keywords:** entomopathogenic fungus, parasitoid, intraguild interaction, integrated pest management, *Tuta absoluta*

## Abstract

**Simple Summary:**

The theory of beneficial species association in a cropping system can sustain ecosystem services and reduce pest pression under economic injury levels. For the control of the invasive pest, *Tuta absoluta* we assessed the susceptibility of *Dolichogenidea gelechiidivoris* to *Metarhizium anisopliae* ICIPE 20 through adult parasitoid and parasitised larval infection; furthermore, we evaluated the preference and performance of sprayed and non-sprayed host plants. We concluded an additive effect for *Tuta absoluta* control by the two biocontrol technologies even though the entomopathogenic fungus reduces the fitness of the parasitoid, such as adult longevity and its performance, and parasitised larval emergence.

**Abstract:**

The Integrated Pest Management (IPM) approach have been widely promoted and used for the management of native and invasive pests, while the use of various components of the IPM can have a synergetic, additive, or antagonistic effect on each other; this study evaluated the susceptibility of *Dolichogenidea gelechiidivoris* (Marsh) (Hymenoptera: Braconidae), to the *Metarhizium anisopliae* (Metschnikoff) ICIPE 20 through direct and indirect infection approaches. The effect of fungus on parasitoid longevity, survival of parasitized-larvae, preference of the parasitoid to fungal treated and untreated larvae, and percent parasitism of *Tuta absoluta* (Meyrick) (Lepidoptera: Gelechiidae) under different infection scenarios were assessed. The direct application of dry conidia to the parasitoid prior to exposure to the host, reduced *D. gelechiidivoris* longevity, though the infected female wasps still yielded high parasitism (over 70%). Infecting the parasitized larvae at different ages led to a respective reduction of parasitoid emergence by 35% and 23% for infection at 1 and 5 days post-parasitisation. Exposure of healthy-*D. gelechiidivoris* adults to a plant-sprayed with fungus did not affect their longevity, and no discriminatory host selection was observed. The highest mortality (~80%) of *T*. *absoluta* was achieved when *D*. *gelechiidivoris* and *M*. *anisopliae* ICIPE 20 were used in combination, indicating an additive impact on the target pest; however, field validation can shed more light on this outcome.

## 1. Introduction

The South American tomato leafminer, *Tuta absoluta* (Meyrick) (Lepidoptera: Gelechiidae) invasion in Afro-Eurasia is a serious threat to tomato (*Solanum lycopersicum* L.) production and livelihoods [1,2,3,4]. Most farmers in sub-Saharan Africa responded to its invasion by using synthetic chemical insecticides as a rapid control method in an attempt to save their tomato crops from this devasting pest [5,6]; however, insecticides, as standalone control tools, are not effective in preventing the pest’s damage to below economic thresholds. More importantly, the overuse of synthetic chemical insecticides results in the evolution of resistance in field populations of the pest [7,8,9]; furthermore, an unrelenting increase in insecticide use is detrimental to human and environmental health because of chemical residues in tomato fruit and disadvantageous effects on non-target organisms, respectively [10,11,12].

Among the safer alternatives to synthetic chemical insecticides, biological control is an ideal option; this control strategy has no risk to human health and other nontargets, as well as being environmentally friendly and can be incorporated with ecofriendly integrated pest management (IPM) tactics [1,13,14,15,16]; this approach is considered an inoffensive method for sustainable agriculture [17,18] with no negative effect on biodiversity, farmers, and consumers [19].

To promote the sustainable management of *T. absoluta* in Africa, the International Centre of Insect Physiology and Ecology (*icipe*) imported the solitary endoparasitoid *Dolichogenidea gelechiidivoris* Marsh (Hymenoptera: Braconidae) from South America, the pest’s native region [20], for classical biological control of this pest. *Dolichogenidea gelechiidivoris* parasitised all larval stages of *T. absoluta* with a preference for the early instar larvae [20]. The parasitoid has the potential to achieve a parasitism rate of up to 86% on *T. absoluta* larvae [21]. *Dolichogenidea gelechiidivoris* was lately released in East African countries (Kenya, Uganda, and Ethiopia) to control *T. absoluta* [22], and significant impact is expected from this parasitoid [23,24].

Within the entomopathogenic arena, several microbial organism were documented to be highly pathogenic to the different life stages of *T. absoluta* [25,26,27,28,29]. For example, Akutse et al. [15] reported *M. anisopliae* isolates ICIPE 18, ICIPE 20, and ICIPE 665 with respective mortalities of 95.0, 87.5, and 86.25% in *T. absoluta* adults. Even higher virulence (100% mortality) of these isolates was reported on the fourth instar [15]. In Africa, several isolates of *M. anisopliae* have been registered and commercialized against various insect pests in several African countries; these include *M. anisopliae* ICIPE 69, targeting fruit flies, mealybugs, and thrips and currently also used for the management of *T. absoluta* [10,30].

The combined application of parasitoids and fungal-based biopesticides may enhance the control of *T. absoluta* and the overall success of IPM programs against this pest. Several findings have demonstrated that entomopathogenic fungi and parasitoids/predators can coexist and manage different insect pest species [31,32,33,34,35]; however, detrimental effects of entomopathogenic fungi on adult or larval survival and other fitness parameters have been reported on some parasitoids [31,36,37,38,39]. The findings by these authors call for a proper understanding of the nature of the interactions between entomopathogenic fungi and other natural enemies before their potential combined use for effective and sustainable control of insect pests. As highlighted above both *D. gelechiidivoris* and *M. anisopliae* ICIPE 20 isolate have been proved to be very promising candidates for biological control of *T*. *absoluta*; however, the nature of interactions between these two biocontrol agents has not been elucidated; this research aims to evaluate the effect of direct infection of *D. gelechiidivoris* with *M. anisopliae* ICIPE 20 and the influence of *M. anisopliae* ICIPE 20 infected host larvae on the behavior and performance of *D. gelechiidivoris*.

## 2. Materials and Methods

### 2.1. Metarhizium anisopliae ICIPE 20 Culture and Viability Assessment

*Metarhizium anisopliae* ICIPE 20 used in this study was acquired from the Germplasm of the Arthropod Pathology Unit, at *icipe*. The fungus was sub-cultured on Sabouraud dextrose agar (SDA) (OXOID CM0041, Oxoid Ltd., Basingstoke, UK), and kept in an incubator at 25 ± 2 °C in full obscurity. From a mother plate, conidiospores were collected by scratching the surface of two-old sporulated cultures using a sterile spatula. The collected conidia were added to 10 mL sterilized distilled water having 0.05% (*w*/*v*) Triton X-100 (MERCK KGaA, Darmstadt, Germany) and vortexed for five min at 700 rpm to guarantee the homogeneity of the suspension. Conidia concentrations were quantified utilizing an improved Neubauer hemacytometer under a light microscope (LEICA DM 2000, Leica Microsystems, Morrisville, NC, USA) as described by Goettel and Inglis [40]. The conidia suspension concentration of 1 × 10^8^ conidia mL^−1^ was obtained through serial dilution. Before performing any bioassay, the viability of the spores was assessed by spread plating 100 µL of the suspension on a SDA plate under a sterile laminar air flow hood. The inoculated plates were hermetically sealed with a Parafilm membrane and kept at 25 ± 2 °C in total darkness. At 18 h post-incubation, lactophenol aniline cotton blue (Millipore Corporation, Billerica, MA, USA) was added into the plates to end the germination procedure and stain the spores to improve their visibility for counting. The germination rate (%) of conidiospores was evaluated from 100 conidia randomly selected using a light microscope (LEICA DM 2000, Leica Microsystems, Morrisville, NA, USA) following the process explained by Goettel and Inglis [40]. Five plates were assessed, and the average percentage germination of the spores was more than 99% viability for all the bioassays.

### 2.2. Insect Rearing

Colonies of *T. absoluta* and *D. gelechiidivoris* were reared and maintained in maintained at the Animal Rearing and Containment Unit (ARCU) at *icipe*.

#### 2.2.1. *Tuta absoluta* Colony

The *T. absoluta* colony was established from tomato leaves infested with larvae collected from a tomato farm in Kirinyaga County, Kenya. The leaves were incubated in a ventilated Perspex cage (50 × 50 × 60 cm). The incubated larvae were supplied with clean tomato leaves, sourced from an insecticide-free screenhouse at *icipe*, until larval pupation and moth emergence. The emerged moths represented the 1st generation of the colony. For colony maintenance, four weeks old potted tomato plants (cv. Money maker), grown in the screenhouse, were placed in a Perspex cage of the same size as that used for incubation. After 48 h, the plants were removed and kept until the eggs hatched. Subsequently, leaves, having early instar larvae, were excised from the plants, and placed in a clean Perspex cage lined with a paper towel to absorb excess moisture, caused by the leaves. The larvae were provided with fresh tomato leaves *ad libitum* as a diet until pupation, and 80% honey-drops were applied on the top of the cage as food for the moths that would emerge. Infested leaves were collected from tomato fields in Kirinyaga every three, or four months and adult moths infused into the colony to rejuvenate genetic vigor and avoid deterioration of the colony due to inbreeding. The colony was maintained at 25 ± 2 °C, 70 ± 5% RH, and a 12L:12D photoperiod.

#### 2.2.2. *Dolichogenidea gelechiidivoris* Colony

The initial cohort of the D. gelechiidivoris colony was obtained from the International Potato Center (CIP) and maintained at *icipe* since 2017. The parasitoid was reared according to the protocol described by Mama Sambo et al. [41]. The colony was maintained at 22 ± 1 °C, 70 ± 5% RH, and 12L:12D photoperiod. Four potted tomato plants with early instar larvae of *T. absoluta* were exposed to a cohort of *D. gelechiidivoris* in a Perspex cage (40 × 20 × 50 cm) for parasitisation. After the exposure period of two days, the plants were removed from the cage and the leaves with parasitized larvae were excised, and then kept in another cage without parasitoids, but lined with paper towel. Fresh tomato leaves (i.e., food source) were added as needed until cocoon formation. *Dolichogenidea gelechiidivoris* adults that emerged were aspirated into a clean cage and fed on 80% honey solution, streaked on the top-interior of the cage.

### 2.3. Effect of M. anisopliae ICIPE 20 on the Performance and the Longevity of D. gelechiidivoris Adults

Three newly emerged *D. gelechiidivoris* couples (3 males:3 females) were infected with 0.5 g dry *M. anisopliae* ICIPE 20 conidia in an infection chamber. The infection chamber consisted of a cylindrical plastic tube (11 × 6 cm), covered on the inside with velvet cloth as described by [42]. Three minutes after exposure to the fungus, the infected parasitoids were removed from the infection chamber, and then released into a clean ventilated Perspex cage (20 × 15 × 14 cm). Sixty first-instar larvae of *T. absoluta* were placed on a fresh tomato stem to mine. The infested stem was then introduced to the infected parasitoids for parasitisation for 24 h. For the control, 60 *T. absoluta* first-instar larvae were exposed to three couples of untreated (without fungus) *D. gelechiidivoris*, which were previously introduced into a fungus-free infection chamber. The larvae were removed after 24 h, incubated and maintained under ambient conditions (25 ± 2 °C and 70 ± 5% RH). Mortality of the exposed *D. gelechiidivoris* was monitored daily and the dead wasps were recorded until the death of all individuals. Pupation of the larvae that were exposed to the fungus-infected parasitoids was recorded, as well as the emergence of either *T. absoluta* adults or parasitoids. One week after the last observed emergence of moths or parasitoids, the remaining cocoons from which nothing emerged, were dissected to reveal pharate adults of either *T. absoluta* or parasitoids. The treatments were set up in a randomised complete block design (RCBD), and the trial was replicated 10 times. Parasitism rate was evaluated as the number of emerged *D. gelechiidivoris* plus the number of dissected cocoons, divided by the sum of the number of *T. absoluta,* the number of pupae that did not emerge, the number of *D. gelechiidivoris* emerged, and cocoons dissected. The sex ratio was expressed as the percentage of females out of the total eclosed *D. gelechiidivoris*.

Cadavers of *D. gelechiidivoris* (parents) and their offspring (F1) were surface disinfected by dipping them in 70% ethanol for 1 min, and then by washing twice in sterilised distilled water. The insects were then put into Petri dishes covered with moist filter papers to assess fungal outgrowth on the cadaver. Petri dishes were tightly sealed with Parafilm and kept at 25 ± 2 °C for 5 days. Death as a result of *M. anisopliae* ICIPE 20 infection was confirmed by the existence of hyphae and conidiospores on the cuticula of the cadaver. A sterile pin was used to collect the fungus from the identified insects and to place it on a glass slide with a droplet of distilled water. The glass slide was observed under an oil immersion microscope and compared with a mother solution of *M. anisopliae* ICIPE 20 to record the mycosis (presence or absence of *M. anisopliae* ICIPE 20 on the incubated insect cadaver). All 30 *D. gelechiidivoris* couples directly infected were incubated and observed for mycosis as well as 30 randomly selected dead individuals from their offspring.

### 2.4. Effect of Metarhizium anisopliae ICIPE 20 on D. gelechiidivoris Larvae

To measure the impact of *M. anisopliae* ICIPE 20 on two immature stages of *D. gelechiidivoris* (egg and larvae), infested tomato leaves with 60, first-instar *T. absoluta* larvae were exposed to three, one-day-old *D. gelechiidivoris* couples for 24 h to ensure parasitisation. Thereafter, the leaves with the exposed larvae were removed from the cage and placed into a plastic container (21 × 15 × 15 cm). Then 10 larvae were randomly selected and removed from the tomato leaves using a camel hair brush and put on a paper towel. Ten (10 mL) of *M. anisopliae* ICIPE 20 suspension at a concentration of 10^8^ conidia/mL was then prepared with sterile distilled water containing 0.05% (*w*/*v*) Triton X-100. Three (3 mL) of the fungal suspension were applied to the 10 larvae on a paper towel. After three minutes on the sprayed paper towel, larvae were incubated and provided with healthy tomato leaves for feeding and development until parasitoid or *T. absoluta* emergence; this setup was considered as the treatment. A similar number of *T. absoluta* larvae parasitized as above were treated with 3 mL sterile distilled water containing 0.05% (*w*/*v*) Triton X-100 and provided with tomato leaves, and this served as the control. The trial was organized in a randomized complete block design (RCBD) and replicated 10 times. The above setup of treatment and control was repeated at five days post parasitisation of *T. absoluta* larvae. The number of eclosed *T. absoluta* and *D. gelechiidivoris* as well as the time taken from parasitisation to emergence of the wasps and their sex were recorded.

### 2.5. Dolichogenidea gelechiidivoris Preference for and Performance on M. anisopliae ICIPE 20-Sprayed and T. absoluta Infested Host Plants

Behavioral activities (landing, walking, resting, probing, and oviposition) of the parasitoid were investigated in no-choice and choice tests to assess the preference of *D*. *gelechiidivoris* to *T. absoluta* infested host plants treated with *M. anisopliae* ICIPE 20. For the choice test, tomato plants were infested with 30 first-instar *T. absoluta* larvae per plant and sprayed with 10 mL of *M. anisopliae* ICIPE 20 suspension at the concentration of 1 × 10^8^ conidia/mL. Another plant infested with 30 first-instar *T. absoluta* larvae was sprayed with 10 mL sterile distilled water containing 0.05% (*w*/*v*) Triton X-100 solution. The two groups of tomato plants were kept for an hour for the suspension to dry and then exposed simultaneously to three mated, one-day-old *D. gelechiidivoris* females in a cage (20 × 15 × 14 cm) for 24 h. Behavioral activities of the parasitoid females, such as landing on the plant, walking, resting, probing, and ovipositing were recorded at five-minute intervals for one hour during their most active time, which is in the morning to midday (from 9:00–12:00 h) (Mama Sambo, personal observation). For the no-choice test, tomato plants with 60 larvae were sprayed with either *M. anisopliae* ICIPE 20 or sterile distilled water containing 0.05% (*w*/*v*) Triton X-100 (control treatment) as described in the choice test. The larvae in both the treatment and control plants were exposed in separate cages to three mated parasitoid females for 24 h. Data were recorded as described for the choice test. In addition, mortality of parasitoid females was also recorded daily. To assess the performance of *D. gelechiidivoris* on *M. anisopliae* ICIPE 20-sprayed and non-sprayed *T. absoluta* infested plants, the plants were incubated separately to record the emergence of *T. absoluta* and *D. gelechiidivoris,* and the sex ratio of F1 progeny of the parasitoid. The cocoons from which no parasitoids emerged were dissected to verify the sex of non-eclosed wasps. The female cadavers from each test (choice test, and the respective no-choice tests) were incubated, and a mycosis test was performed as described above in Section 2.1. Thirty couples were also selected from the offspring of each of the tests to perform a mycosis test.

### 2.6. Efficiency of D. gelechiidivoris, M. anisopliae ICIPE 20 and Their Combination on T. absoluta

Data from the above bioassay in a no-choice test was considered to evaluate the percentage emergence of *T. absoluta*. Additionally, a positive control with only *M. anisopliae* ICIPE 20 applications on an infested host with 60 fist-instar larvae and negative control with only distilled water containing 0.05% (*w*/*v*) Triton X-100 application was settled. *Tuta absoluta* percentage of emergence for the different sets of bioassays was compared.

### 2.7. Data Analysis

The Kaplan–Meier estimator method was used to estimate the survival function for parasitoid longevity data. Since the behavioral activities were assessed repetitively, normally assumption of parametric tests was violated [43,44], and repeated-measures ANOVA were run to differentiate between the number of activities on *M. anisopliae* ICIPE 20 sprayed and that on non-sprayed host plants in the choice and no-choice tests. Emergence data were analyzed using GLM with a negative binomial distribution, and developmental time data using GLM with a gamma distribution. When significant differences were noticed, multiple means comparisons were done using Tukey’s HSD test, at α = 0.05. Statistical analyses were performed using R 3.5.1 [45].

## 3. Results

### 3.1. Effect of M. anisopliae ICIPE 20 on the Performance and the Longevity of D. gelechiidivoris Adults

**Figure 1:**
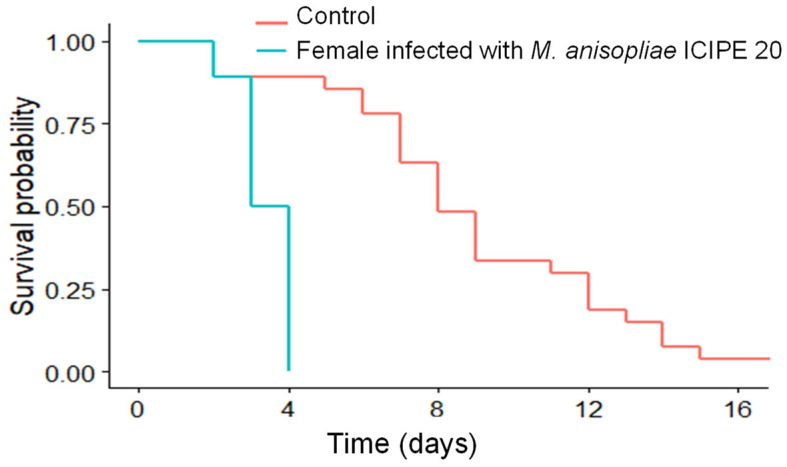
Effect of *M. anisopliae* ICIPE 20 (direct infection with dry conidia) on *D. gelechiidivoris* survival.

The survival time of *D. gelechiidivoris* was significantly reduced (*p* < 0.001) whereby 50% wasps died by day three post-infection with dry *M. anisopliae* ICIPE 20 conidia (treatment), while uninfected wasps had longer longevity with up to 8 days median survival time (Figure 1). The reduced survival of infected wasps was confirmed being caused by the fungus and 90% of adult parasitoid cadavers showed mycosis.

**Figure 2:**
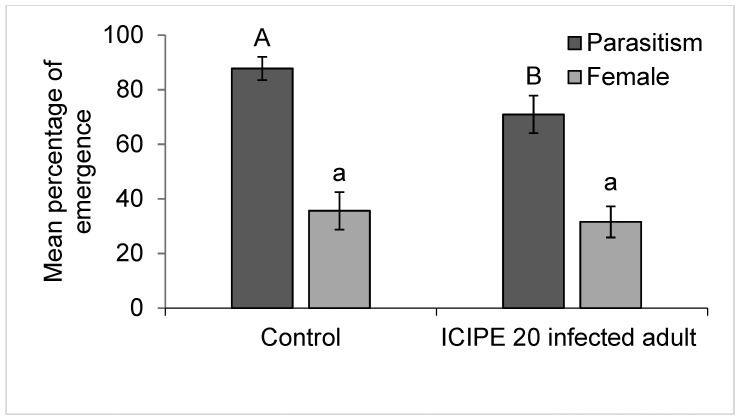
Performance of *M. anisopliae* ICIPE 20 infected and uninfected *D. gelechiidivoris* on *T*. *absoluta*. Bars capped with the same upper/lower case letters are not significantly different.

The performance of *M. anisopliae* ICIPE 20 infected and uninfected *D. gelechiidivoris* females was measured as percent parasitism of *T*. *absoluta,* It varied between treatments (F_1,18_ = 4.88, *p =* 0.040), and was significantly higher (87.79 ± 6.32%) for uninfected wasps. Nonetheless, no transmission of ICIPE 20 conidia by infected *D. gelechiidivoris* parents to offspring was observed. There was also no significant difference between the sex ratio of offspring of infected and uninfected wasps (F_1,18_ = 0.207, *p* = 0.65), in both cases the sex ratio was male bias (Figure 2).

### 3.2. Effect of M. anisopliae ICIPE 20 on the Development of Parasitised T. absoluta Larvae

The developmental time of the immature stages of *D. gelechiidivoris* was not affected by the fungal application to *T. absoluta* larvae one day post-exposure to the parasitoid, for both male (F_1,10_ = 0.97, *p =* 0.35) and female (F_1,13_ = 0.34, *p =* 0.60). The developmental time of males was different for the untreated and treated larvae (F_1,15_ = 7.38, *p =* 0.01). Conversely, female developmental time was comparable between fungal treated and untreated larvae (F_1,19_ = 0.65, *p =* 0.43), being shorter for treated larvae (Table 1).

**Figure 3:**
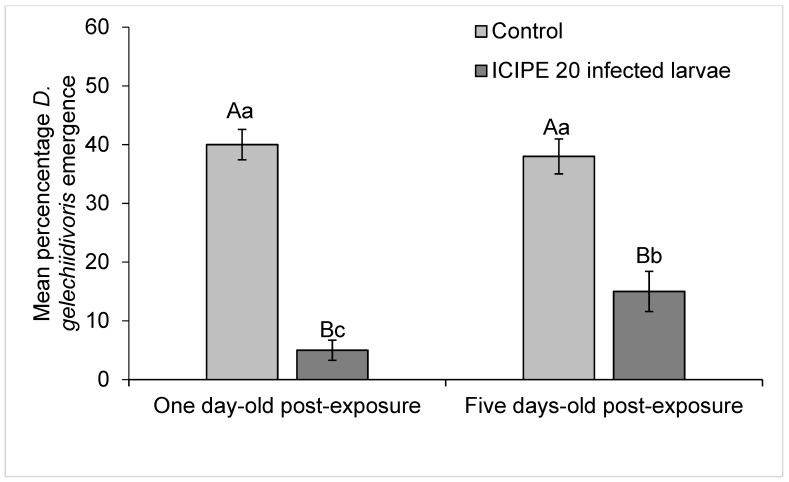
Percentage of *D.* *gelechiidivoris* that emerged from larvae exposed to the parasitoids infected with *M. anisopliae* ICIPE 20 at one day and five days post-exposure. Bars capped with the same uppercase letter indicate no difference between control and treatment within the same age exposed-larvae, bars capped with the same lowercase letter indicate no difference between one day and five days post-exposure.

The percent eclosed *D*. *gelechiidivoris* wasps varied between *M. anisopliae* ICIPE 20 infected and non-infected larvae for both one day old post-exposure (F_1,18_ = 18.15, *p <* 0.001) and five days old post-exposure (F_1,18_ = 26.3, *p <* 0.001), being lowest (5 ± 1.67%) for one day old post-exposure to the fungus (Figure 3).

### 3.3. Dolichogenidea gelechiidivoris Preference for and Performance on M. anisopliae ICIPE 20-Sprayed and T. absoluta Infested Host Plants

The preference of *Dolichogenidea gelechiidivoris* to fungal sprayed and non-sprayed plants as measured by the different behavioral response of foraging females was not affect by the host plant status (Table 2).

**Figure 4:**
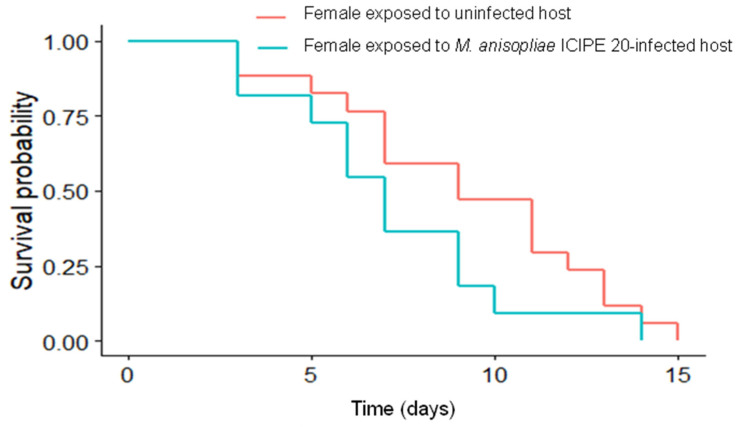
Survival of *D. gelechiidivoris* female foraging on *Metarhizium anisopliae* ICIPE 20 sprayed and unsprayed host plants.

**Figure 5:**
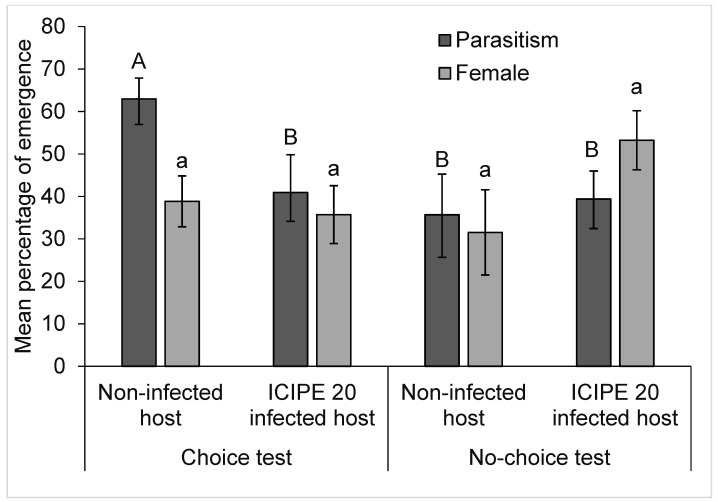
Performance of *D. gelechiidivoris* on *M. anisopliae* ICIPE 20 sprayed and unsprayed plants Bars capped with same upper/lower case letters are not significantly different.

The longevity of *D.* *gelechiidivoris* females foraging on fungal-infected and uninfected host plants did not differ significantly (*p* = 0.14). The median survival time of females exposed to infected and uninfected hosts was nine and eight days, respectively (Figure 4). Furthermore, only 43% of *D. gelechiidivoris* cadavers from larvae in plants that received an *M. anisopliae* ICIPE 20 application were found to have mycosis Percent parasitism of *T*. *absoluta* by *D.* *gelechiidivoris* differed between *M. anisopliae* ICIPE 20 sprayed and non-sprayed plants in choice test (F_1,18_ = 4.68, *p* = 0.044), being higher (62%) on the latter. While in the no-choice test, there was no significant difference (F_1,18_ = 0.10, *p* = 0.75); however, the proportion of females that emerged from parasitised larvae did not differ significantly between *M. anisopliae* ICIPE 20 sprayed and non-sprayed plants in the choice (F_1,18_ = 0.12, *p =* 0.73), as well as in no-choice scenario (F_1,18_ = 3.16, *p* = 0.09) (Figure 5). From the F1 of the offspring of *D. gelechiidivoris* that foraged on fungal sprayed host plants, 77% were infected with *M. anisopliae* ICIPE 20.

### 3.4. Efficiency of D. gelechiidivoris, M. anisopliae ICIPE 20 and Their Combination on T. absoluta

**Figure 6:**
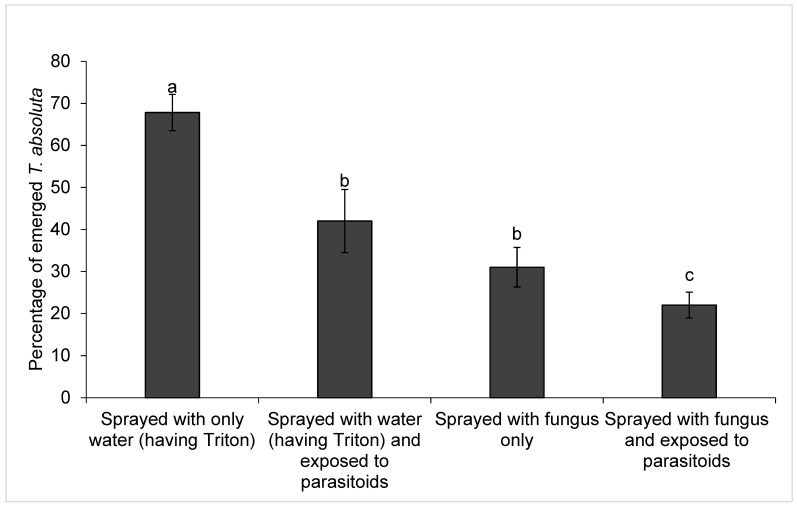
Effect of sole and combined use of two biocontrol agents on *Tuta absoluta* emergence. Bar capped with the same letters are not significantly different, (Tukey’s HSD, α = 0.05).

The percent emerged moths of *T. absoluta* varied considerably (F_3,36_ = 14.56, *p* ˂ 0.001) among the treatments (when exposed to *D. gelechiidivoris* alone, subjected to infection by *M. anisopliae* ICIPE 20, and subjected to the fungal infection followed by exposure to the parasitoid and untreated control plants). More *T. absoluta* adults emerged from the untreated control plants, while the lowest emergence was observed from plants exposed to *D. gelechiidivoris* that were previously sprayed with *M. anisopliae* ICIPE 20 (Figure 6).

## 4. Discussion

Several *Metarhizium* species and strains have been reported to be pathogenic to different life stages of *T. absoluta* [15,25,46,47,48]. Different strategies of using these fungal isolates including their integration with pheromone traps using dry conidia through an autodissemination method [15], and inundative application of the fungus as direct sprays [28,49] have been demonstrated for the control of *T. absoluta*. When several management strategies such as the use of parasitoids, entomopathogens and mass trapping are implemented in the context of IPM, parasitoids could be attracted to or accidentally enter the *T. absoluta* trap impregnated with *M. anisopliae* ICIPE 20, thus resulting in detrimental effects to the functioning of the parasitoids. Furthermore, although parasitoids may avoid infected hosts, they can be in contact with the inoculum and their survival inside or on the infected host may be hampered.

In the current study, infecting *D. gelechiidivoris* directly with dry conidia of *M. anisopliae* ICIPE 20 reduced percent parasitism as well as the longevity of the parasitoid. Similar results were reported for other parasitoid species. For example Nozad-Bonab et al. [50] working on the same host used in this study, found that infecting *Trichogramma brassicae* Bezdenko (Hymenoptera: Trichogrammatidae) with *M. anisopliae*, reduced the longevity of the parasitoid. Similarly, in a study by Presa-Parra et al. [51] the longevity of *M. anisopliae* infected Diachasmimorpha longicaudata Ashmead (Hymenoptera: Braconidae) was much shorter compared to their untreated counterparts. *Dolichogenidea gelechiidivoris* lays the highest number of eggs at one to three days after female emergence [20], when the parasitoid was *infected* with *M. anisopliae* ICIPE 20 due to the attraction to *T. absoluta* pheromone traps as found by Ayelo et al. [52]. Considering the findings by these authors and in the light of the result of this study that the median survival time of fungus-infected females were 3 days coupled with the fact that they cause a parasitism level of more than 70%, the parasitoid population growth might not be significantly affected by the fungal application. Our result of percent mycosed wasps (90%) following infection with dry *M. anisopliae* ICIPE 20, was different from that (50%) reported by Nielsen et al. [31] for the same fungus when tested on *Spalangia cameroni* Perkins (Hymenoptera: Pteromalidae). The discrepancy between our results and that by Nielsen et al. [31], could be due to the fact that these authors used fungal conidial suspensions while we used dry conidia. Another possible explanation could be due to difference in the fungal isolate used in the two studies.

We have demonstrated that infecting the *T. absoluta* parasitized larvae *with M. anisopliae* ICIPE 20 reduced the survival of immature stages of *D. gelechiidivoris*, with the egg stage more affected than the larval stage. Using the same fungus different levels of pathogenicity against *T. absoluta* have been documented. For example, Contreras et al. [28] reported more than 80% pathogenicity to pupae of *T. absoluta* from different populations, while Rodríguez et al. [53] reported more than 90% mortality of third instar *T. absoluta larvae,* and Akutse et al. [15] found 100% mortality of fourth instar *T. absoluta* larvae. The differential survival of the immature stages of *D. gelechiidivoris* reared on fungal infected host larvae could be due to the fact that parasitoid larvae release fungicidal substances inside the host to stop fungus growth, thus facilitating the development of their offspring as argued by Fransen and van Lenteren, [54]. Although the survival of the immature stages of *D. gelechiidivoris* was reduced by fungal treatment of their host, interestingly in general the developmental time of the eclosed wasps was not affected by the fungal treatment. Similarly, Ramos Aguila et al. [55] found that the developmental time of *Tamarixia radiata* Waterston (Hymenoptera: Eulophidae) reared on *Beauveria bassiana* infected Diaphorina citri Kuwayama (Hemiptera: Liviidae) was similar to the parasitoids being reared on healthy hosts.

With regard to *D. gelechiidivoris* preference, the female wasps could not distinguish between *M. anisopliae* ICIPE 20-infected and uninfected hosts, nonetheless high percent parasitism was recorded on an uninfected host in choice experimental conditions. Similarly, non-discriminatory behavior was also reported for the parasitoid, *Aphelinus asychis* Walker (Hymenoptera: Aphelinidae) exposed to *Diuraphis noxia* Kurdjumov (Hemiptera: Aphididae) populations infected with the fungus *Isaria fumosorosea* (Paecilomyces) compared to uninfected host [56]. The non-discrimination behavior of *D. gelechiidivoris* between infected and uninfected hosts could be due to the absence of odor of *M. anisopliae*, as reported in the case of mosquitoes species [57]. Additionally the response of a species involves a co-evolutionary phenomenon [58] and could explained the non-discriminatory behavior of *D. gelechiidivoris* towards the fungal-infected and uninfected host. Contrary to our findings, *Trichogramma pretiosum* Riley (Hymenoptera: Trichogrammatidae) discriminated against the *M. anisopliae* infected eggs of *Anagasta kuehniella* Zeller (Lepidoptera: Pyralidae) when given a choice [34]. Similarly, Miranda-fuentes et al. [38] found that the cotton leafworm, *Spodoptera littoralis* (Boisduval) (Lepidoptera: Noctuidae) infected with *Metarhizium brunneum* Petch (Hypocreales: Clavicipitaceae) was less preferred by the endoparasitoid, *Hyposoter didymator* (Thunberg) (Hymenoptera: Ichneumonidae) with parasitism being almost three times higher in the uninfected compared to the infected larvae.

In terms of parasitoid emergence, a lower parasitism rate was recorded on the sprayed host plant in a choice test, suggesting that the fungus might have affected the growth and development of the parasitoid. In a previous study by Potrich et al. [34], the eggs of *Anagasta kuehniella* Zeller (Lepidoptera: Pyralidae) infected with *Metarhizium anisopliae* Unioeste 43 and *M. anisopliae* ESALQ 1641 were 44% and 41%, respectively less parasitised compared to the healthy host when offered to *T. pretiosum* in a choice test. On the other hand, spraying eggs of *Duponchelia fovealis* (Zeller) (Lepidoptera: Crambidae) with commercial *M. anisopliae* IBCB348 at 1.5 × 105 conidia mL^−1^ did not affect the choice of *Trichogramma atopovirilia* Oatman and Platner and *T. pretiosum* (Hymenoptera: Trichogrammatidae) to parasitise and no effect on the parasitoid emergence, or sex ratio of the progeny was reported [59]. Furthermore, Domingues et al. [39] reported comparable parasitism rates by the parasitoid *Cleruchoides noackae* Lin and Huber (Hymenoptera: Mymaridae) between infected and uninfected eggs of *Thaumastocoris peregrinus* Carpintero and Dellapé (Hemiptera: Thaumastocoridae) treated with *M. anisopliae*. The simultaneous release of the parasitoid *D. gelechiidivoris* and the spraying of *M. anisopliae* will therefore have limited effects on the parasitoid since a choice scenario might be observed in real conditions.

The highest level of *T. absoluta* mortality occurred with the combined use of the two biocontrol agents, whereby *M. anisopliae* ICIPE 20 was applied to an infested plant followed by the release of *D. gelechiidivoris.* In all scenarios, when the fungus was applied first, and the parasitoid encountered an already infected host, longevity and parasitism were not affected. Against this background, we envisage that under field conditions, with application of *M. anisopliae* ICIPE 20 prior to the release of the parasitoid, the likelihood of the fungus negatively impacting on the parasitoid performance will be negligible. Similarly Nozad-Bonab et al. [50] reported 94–95% mortality of *T. absoluta* when *T. brassicae* was used in combination with *M. anisopliae*. In contrast, Presa-Parra et al. [51] using the same fungus found that there was no difference in mortality of parasitised and non-parasitised *Anastrepha ludens* Loew (Diptera: Tephritidae) larvae sprayed with *M. anisopliae*.

## 5. Conclusions

Although direct infection of *D. gelechiidivoris* adults with *M. anisopliae* ICIPE 20 reduced the longevity of the parasitoid, the infected females were able to achieve a considerable level of parasitism (more than 70%). The direct infection of *D. gelechiidivoris* adults did not result in any infection of the offspring. The study also demonstrated that mortality of the parasitoid following the infection of host larvae was a function of the time at which the host was subjected to *M. anisopliae* ICIPE 20 infection, with more time between parasitism and fungal infection resulting in lower mortality of the parasitoid. With regard the potential use of the two biocontrol agents evaluated in this study, sequential use *D. gelechiidivoris and* *M. anisopliae* ICIPE 20 yielded the highest mortality of the host, suggesting and additive effect on the target pest. Therefore, the combination of *M. anisopliae* ICIPE 20 application and *D. gelechiidivoris* for management of *T. absoluta* can offer a promising alternative to chemical control applications. However, field or semi-field trials on the combined use of these two important biocontrol agents (*D. gelechiidivoris* and *M. anisopliae* ICIPE 20) will shed more light on their performance in suppression of *T*. *absoluta*, a study which we are currently undertaking.

## Figures and Tables

**Table 1:** Developmental time (Mean ± SE) of *D.* *gelechiidivoris* on *M. anisopliae* ICIPE 20 treated and untreated *T*. *absoluta* larvae.

Age Before Exposure	Treatments	No. Days ± SE (♂)	No. Days ± SE (♀)
One day	Control	18.8 ± 0.99 a	19.58 ± 1.02 a
	Treatment	16 ± 1.5 a	18.33 ± 0.88 a
Five days	Control	21.33 ± 0.85 a	25.19 ± 4.21 a
	Treatment	17.40 ± 0.75 b	19.0 ± 1.14 a

Means with the same letter within a column are not significantly different (Tukey’s HSD, α = 0.05).

**Table 2:** Number of behavioral activities (means ± SE) performed by three females of *D. gelechiidivoris*/5 min on sprayed and non-sprayed host plant in choice and no-choice tests.

Behavioral Activities	Choice Test			No-Choice Test		
	Means ± SE		Statistics	Means ± SE		Statistics
	Non-Sprayed	Sprayed		Non-Sprayed	Sprayed	
Landing	1.10 ± 0.25	0.63 ± 0.15	F_1,58_ = 2.57, *p* = 0.11	0.50 ± 0.14	0.63 ± 0.16	F_1,58_ = 1.59, *p* = 0.21
walking	1.50 ± 0.30	1.00 ± 0.31	F_1,58_ = 1.30, *p* = 0.26	1.43 ± 0.49	0.77 ± 0.42	F_1,58_ = 0.32, *p* = 0.57
Resting	0.9 ± 0.2	0.67 ± 0.16	F_1,58_ = 0.49, *p* = 0.49	0.57 ± 0.23	0.83 ± 0.25	F_1,58_ = 1.07, *p* = 0.30
Probing	5.33 ± 1.10	3.27 ± 1.10	F_1,58_ = 2.08, *p* = 0.15	4.77 ± 0.88	2.80 ± 1.20	F_1,58_ = 1.72, *p* = 0.19
Oviposition	0.36 ± 0.13	0.33 ± 0.17	F_1,58_ = 0.02, *p* = 0.88	0.67 ± 0.31	1.03 ± 0.30	F_1,58_ = 0.72, *p* = 0.40

## Data Availability

The data of these results is available on the International Centre of Insect Physiology and Ecology (*icipe*) data repository at the link: https://dmmg.icipe.org/dataportal/dataset/interactions-between-the-entomopathogenic-fungus-metarhizium-anisopliae-icipe-20.
